# The Influence Path Analysis of Clinical Nurses’ Job Satisfaction Based on the Dual‐Factor Theory

**DOI:** 10.1155/jonm/7864879

**Published:** 2026-05-12

**Authors:** Wanning Jia, Haiyan Wang, Liqian Gao, Zhuozhuo Chen, Haitao Lu, Wan Dong

**Affiliations:** ^1^ Blood Purification Center, China-Japan Friendship Hospital, No. 2 East Yihua Street Chaoyang District, Beijing, China, zryhyy.com.cn

**Keywords:** clinical nurse, job satisfaction, missed anxiety, qualitative comparative analysis, two-factor theory

## Abstract

**Background:**

Clinical nurses significantly contribute to sustaining healthcare systems worldwide. However, they experience multiple adverse situations that affect their job satisfaction, which in turn influences the quality of nursing services.

**Aim:**

To analyze the pathways influencing clinical nurses’ job satisfaction from the perspective of the dual‐factor theory.

**Methods:**

We recruited 202 clinical nurses from August to September 2024 using a convenience sampling method for this cross‐sectional questionnaire survey assessing job burnout, job satisfaction, and fear of missing out. The fuzzy‐set qualitative comparative analysis (fsQCA) method was used to examine the factors, their synergistic effects, and the configurational pathways through which different combinations of factors influence clinical nurses’ job satisfaction.

**Results:**

The average score for job satisfaction in this study was 60.28 ± 10.51 points. Single‐factor analysis showed that age, fear of missing out, and job burnout were significantly associated with job satisfaction. However, fsQCA necessity analysis indicated that no single factor was a necessary condition for high job satisfaction (consistency < 0.9). The configuration analysis extracted six sufficient condition combinations, categorized into two types: hygiene‐dominated and motivation‐health configurations. Among these, age (as a moderating variable) emerged as a core condition in several configurations.

**Conclusion:**

The fsQCA method, grounded in the two‐factor theory, illustrated the influence of multiple interacting and synergistic factors on clinical nurses’ job satisfaction and identified the specific elements contributing to high job satisfaction within various configurations of their roles. This insight helps hospitals and nursing managers identify optimal combination of factors that enhance the stability of clinical nurses’ careers and clarify potential improvement pathways from a configuration perspective. Overall, our findings provide novel evidence and a framework for the development of effective interventions to enhance job satisfaction.

## 1. Introduction

Nurses play a crucial role in clinical nursing practice, and their working conditions directly influence the quality of nursing services [1]. As a unique group of medical professionals with the longest continuous contact with patients, nurses are increasingly exposed to work‐related pressures, resulting in anxiety and burnout. Continued exposure to such pressures may reduce work enthusiasm and lead to the gradual emergence of symptoms of job burnout. This deterioration in professional engagement can result in decreased job satisfaction and, ultimately, contribute to increased turnover rates among nursing staff [2, 3]. Job satisfaction—defined as employees’ emotional experiences toward their professional environment, working conditions, and work content—is a crucial indicator of subjective attitudes and a key predictor of turnover intention [4, 5]. Therefore, understanding the determinants of job satisfaction for nurses is essential to maintain a stable and engaged nursing workforce.

Previous studies examining the factors influencing job satisfaction in nurses have primarily concentrated on the independent effects of various determinants, such as demographic characteristics, work stress, or organizational support, often using traditional regression‐based methods [6]. Although valuable, these approaches are limited in their ability to capture the comprehensive impacts of multiple interacting factors or the synergistic effects that arise from their combinations [7]. In real‐world clinical settings, job satisfaction in nurses is rarely attributable to a single cause; rather, it emerges from a combination of individual, interpersonal, and organizational conditions. This methodological gap is particularly pertinent in nursing management research, where understanding causal complexity is essential for designing effective interventions.

The fuzzy‐set qualitative comparative analysis (fsQCA) method addresses this limitation by integrating qualitative and quantitative logics to identify sufficient condition combinations that lead to an outcome of interest [8]. Unlike traditional variable‐centered approaches, fsQCA is case‐oriented and examines how different conditions combine to produce an outcome, offering a holistic perspective on causal complexity. Although fsQCA has been increasingly applied in organizational behavior, public health, and health services research [7], its application in nursing workforce studies remains scarce. Given the inherently multifactorial nature of job satisfaction in nurses, fsQCA offers a novel approach to identify configurational pathways often missed via traditional models.

Herzberg’s two‐factor theory, also referred to as “incentive‐health theory” [9–11], posits that work attitudes are shaped by two distinct sets of factors: motivators (e.g., achievement, recognition, and responsibility) that directly enhance satisfaction when present and hygiene factors (e.g., working conditions, interpersonal relations, and organizational policies) whose absence leads to dissatisfaction, but presence does not necessarily generate satisfaction. According to Herzberg, improving hygiene factors can prevent dissatisfaction; however, true work motivation and satisfaction result from addressing motivators. This framework has been widely applied in organizational psychology and human resource management and maybe useful for understanding nurse job satisfaction.

This study aimed to analyze the pathways influencing job satisfaction among clinical nurses from the perspective of the dual‐factor theory. In this study, we operationalized Herzberg’s framework as follows: emotional exhaustion and depersonalization—two core dimensions of job burnout—were conceptualized as hygiene factors, reflecting the depletion of psychological resources and interpersonal distancing due to adverse work environments. Personal accomplishment, the third dimension of burnout, is considered a motivator, representing nurses’ intrinsic sense of competence and meaningful achievement in their work. Age is included as a moderating variable, as it may influence both sensitivity to hygiene factors and the salience of motivators across career stages.

With the widespread adoption of mobile communication and social media in recent years, healthcare professionals frequently engage with digital platforms beyond working hours, a phenomenon that may contribute to the emergence of “fear of missing out” (FOMO). FOMO refers to the pervasive anxiety that one might miss important information, rewarding experiences, or career opportunities that others are having [12]. In clinical nursing, this construct encompasses both a social–psychological dimension and a profession‐specific dimension.

The social–psychological aspect reflects nurses’ concerns about missing out on personal social interactions, leisure activities, or general life events due to demanding shift schedules. In contrast, the professional/clinical dimension—which is the primary focus of this study—manifests as two distinct but related anxieties: professional information anxiety and social isolation during long shifts. Nurses working in high‐intensity, shift‐based environments often worry about missing critical medical updates, new evidence‐based practices, or training and promotion opportunities because of irregular schedules or heavy workloads. Additionally, prolonged patient care duties and rotating schedules may limit their ability to stay connected with colleagues, friends, or family, exacerbating feelings of being “out of the loop” both professionally and personally.

Within Herzberg’s two‐factor framework, FOMO can be categorized as a hygiene factor. When nurses experience unaddressed information gaps or social disconnection, this leads to dissatisfaction with the work environment—a typical hygiene factor dynamic. Failure to manage FOMO appropriately may lead to a build‐up of employee dissatisfaction, thereby reducing job satisfaction. Moreover, FOMO may undermine work engagement and professional identity, indirectly weakening the impact of intrinsic motivators (e.g., recognition and career growth) on job satisfaction. Therefore, FOMO represents a contemporary psychosocial variable that potentially influences nurses’ work attitudes, warranting integration into the two‐factor framework and empirical examination in the clinical nursing context.

Against this backdrop, the present study seeks to address the following research questions: which motivators and hygiene factors, in addition to the moderating variable, are necessary to achieve high job satisfaction among clinical nurses? and how do these configurational pathways align with, or extend, the predictions of Herzberg’s two‐factor theory?

By employing fsQCA grounded in the two‐factor theoretical model, this study aimed to move beyond variable‐centered analyses and provide a holistic, configurational understanding of the job satisfaction for clinical nurses. The findings are expected to make two key contributions. Theoretically, this study will extend the two‐factor theory by specifying how its components combine in real‐world, configurational pathways—revealing synergistic interactions and buffering effects that traditional additive models cannot capture. Practically, the identification of distinct pathways to high job satisfaction will enable nursing managers to design pathway‐specific, tailored interventions that address the unique combination of factors affecting different nurse subgroups, rather than relying on one‐size‐fits‐all approaches. Ultimately, this configurational perspective offers new evidence and analytical frameworks for developing effective strategies to enhance nurse job satisfaction, workforce stability, and patient care quality.

## 2. Materials and Methods

### 2.1. Participants

This study was approved by the Ethics Committee of China‐Japan Friendship Hospital (Approval No. 2023‐KY‐278). All participants provided written informed consent prior to data collection. Between August and September 2024, clinical nurses from a Class III Grade A hospital in Beijing who met the specified inclusion and exclusion criteria were selected to answer questionnaire survey on factors related to job satisfaction using convenience sampling.

The inclusion criteria were as follows: (i) possession of a valid nurse professional qualification certificate, (ii) engagement in clinical nursing practice, and (iii) at least 1 year of work experience. The exclusion criteria were as follows: (i) involvement in advanced study or training programs and (ii) absence during the data collection period due to study or vacation.

### 2.2. Sample Size Estimation

The required sample size was estimated based on the number of items in the survey questionnaires, following the conventional guideline that the sample size should be 10–20 times the number of items [13]. In this study, the survey instruments comprised 15 items/variables: nine items in the general information questionnaire, three dimensions in the job burnout inventory (emotional exhaustion, depersonalization, and reduced personal accomplishment, each treated as a separate variable in the analysis), two dimensions in the job satisfaction scale (work state and work interpersonal relationships), and one dimension in the missed anxiety scale (FOMO). Accordingly, the required sample size was 150–300. Ultimately, 202 questionnaires were distributed to minimize invalid questionnaires and errors.

### 2.3. Survey Methods

#### 2.3.1. General Information Questionnaire

The researcher‐designed questionnaire included questions on nine items: sex, age, marital status, whether having children, education level, intensive care unit (ICU) nursing experience, professional title, position, and years of work experience.

#### 2.3.2. Missed Anxiety Scale

The missed anxiety scale by Przybylski et al. [12] and the Chinese version of the scale revised by Qi [14] according to local Chinese culture were adopted in this study. The revised version comprises 10 items with a one‐way dimension and is scored using a 5‐point Likert scale, where “1 represents never;” “2, rarely;” “3, sometimes;” “4, often;” and “5, always.” The total score ranges from 10 to 50 points. The higher the sum of all items, the higher is the level of miss anxiety. This scale was originally used to assess anxiety related to missing out on social media; however, its core construct, the anxiety of missing important information, is also applicable for evaluating the anxiety of clinical nurses in high‐pressure work environments regarding “information loss” or “missed career development opportunities.” Through preliminary interviews and a pilot survey with clinical nurses, this study confirmed that the scale exhibited satisfactory psychometric properties (Cronbach’s α = 0.873) and conceptual relevance in the clinical nursing context, supporting its cultural and professional appropriateness for the Chinese nursing population [15].

In this study, FOMO was conceptualized as a hygiene factor, reflecting the anxiety experienced by nurses when they feared missing critical information or opportunities.

#### 2.3.3. Burnout Inventory

The Chinese Maslach Burnout Inventory (CMBI) [16] includes the three dimensions of emotional exhaustion, depersonalization, and a reduced sense of personal accomplishment, with a total of 15 items. The items are scored on a 7‐point Likert scale, wherein the scores range from 1 to 7 points indicating completely inconsistent to completely consistent; the score of reduced sense of achievement is reversed. The total score ranges from 15 to 150 points, with higher scores indicating more serious job burnout. The Cronbach’s α coefficient of the scale was 0.816 [17].

Emotional exhaustion and depersonalization were categorized as hygiene factors, reflecting the depletion of psychological resources caused by the work environment, whereas reduced personal accomplishment was associated with motivators, indicating a deficit in intrinsic work motivation.

#### 2.3.4. Job Satisfaction Scale

The content validity of nurse’s working satisfaction scale revised by Hong [18] was 0.890, with a Cronbach’s α coefficient of > 0.800. The scale comprises two dimensions: working status and interpersonal relationships at work, with a total of 15 items scored on a 5‐point Likert scale. Scores range from 1 (very dissatisfied) to 5 (very satisfied), with the total score ranging from 15 to 75 points and higher scores indicating higher job satisfaction.

Job satisfaction was the outcome variable, and its two dimensions—work state and work interpersonal relationships—corresponded to the integrated manifestations of hygiene factors and motivators, respectively.

### 2.4. Constructing a Two‐Factor Theoretical Model Framework for Job Satisfaction of Clinical Nurses

**FIGURE 1:**
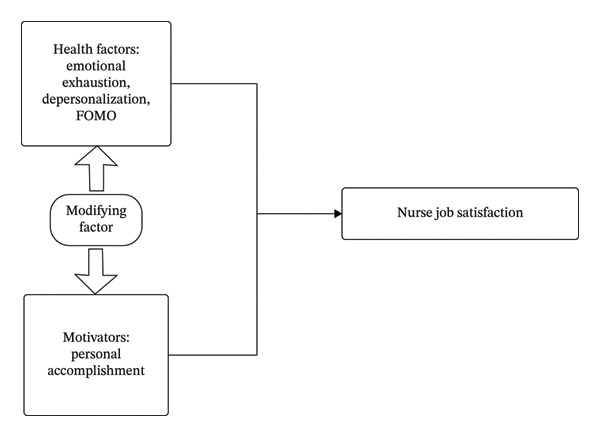
Framework of two‐factor theoretical model for job satisfaction of clinical nurses.

The two‐factor theory aims to help managers identify strategies to improve nurses’ contentment and job satisfaction to foster work enthusiasm [19]. This framework encompasses two key aspects: motivating and health‐related factors. Motivating factors pertain to the intrinsic incentives associated with work and are referred to as incentive factors, which can exert a direct motivational influence on employees. Conversely, health factors are related to elements that may lead to employee dissatisfaction. When health factors are not adequately addressed, they can easily result in feelings of discontent, negative work attitudes, and even confrontational behaviors, such as strikes. The two‐factor theoretical framework of clinical nurses’ job satisfaction is illustrated in Figure 1.

Based on Herzberg’s two‐factor theory, the variables in this study were categorized into (i) hygiene factors, (ii) motivators, and (iii) age. Hygiene factors included emotional exhaustion, depersonalization, and FOMO. A failure to address these factors may cause job dissatisfaction and burnout among nurses. Motivators refer to personal accomplishment, a construct associated with the intrinsic value of work and recognition of achievements, that directly enhances job satisfaction. Age was treated as a moderating (control) variable, as it may exert effects across both factor types.

### 2.5. Survey Methods

After obtaining informed consent from the respondents, the researchers generated a two‐dimensional code for the online questionnaire. During questionnaire distribution, the department’s responsible personnel coordinated to ensure that all participants received guidance simultaneously and provided a unified explanation of the research objectives, data confidentiality, and instructions for completing the questionnaire. Verification questions were incorporated into the questionnaire to ensure that the respondents met the sampling criteria. The completion status of each online submission and overall completeness of the responses were subsequently reviewed by the research team leader. A total of 210 questionnaires were distributed and 202 valid responses were received, resulting in an effective response rate of 96.19%.

### 2.6. Statistical Analysis

SPSS 29.0 (IBM Corp., Armonk, NY, USA) was used for data processing and statistical analysis. Count data are expressed as percentage (%) and were analyzed using *χ*2 test. Measurement data are expressed as mean ± standard deviation and were analyzed using the independent samples *t*‐test.

fsQCA refers to the relationship between the outcome and all possible Boolean combinations of influencing factors. The optimal combination was evaluated using two indicators: “consistency” and “coverage”. First, the calibration (x, n1, n2, and n3) function in the fsQCA software was used to calibrate the raw data. Second, before the condition configuration analysis, the “necessity” of each condition was tested one by one. Consistency and coverage rates were calculated to assess whether a relationship existed between necessity and adequacy. A consistency score > 0.9 indicates a necessary condition. Third, a truth table was constructed with a consistency threshold of 0.80 and a frequency threshold of 1 to perform configuration analysis. Finally, all factors influencing the outcome variables were combined.

## 3. Results

### 3.1. General Information

Table 1 shows general characteristics of the clinical nurses. A total of 202 clinical nurses were surveyed, including 13 males (6.40%) and 189 females (93.60%). Among the participants, 142 (70.30%) were married and 60 (29.70%) were unmarried. Regarding educational background, 17 individuals (8.42%) had a junior college degree, whereas 185 (91.58%) had a bachelor’s degree or higher (Table 1).

**TABLE 1:** Single‐factor analysis of job satisfaction of clinical nurses with different characteristics (*n* = 202).

Items	Low level of job satisfaction group (*n* = 39)	Medium–high level of job satisfaction group (*n* = 163)	Statistics	*p*
Sex (first name, %)			*χ* ^2^ = 0.137	0.711
Male	2 (5.13)	11 (6.75)		
Female	37 (94.87)	152 (93.25)		
Age (first name, %)			*χ* ^2^ = 5.077	0.024^∗^
≤ 40 years old	26 (66.67)	135 (82.82)		
> 40 years old	13 (33.33)	28 (17.18)		
Marital status (first name, %)			*χ* ^2^ = 3.198	0.074
Unmarried	7 (17.95)	53 (32.52)		
Married	32 (82.05)	110 (67.48)		
Presence or absence of children (name, %)			*χ* ^2^ = 0.009	0.925
Absent	34 (87.18)	143 (87.73)		
Present	5 (12.82)	20 (12.27)		
Education (name, %)			*χ* ^2^ = 2.147	0.143
Junior college	1 (2.56)	16 (9.82)		
Bachelor’s degree and above	38 (97.44)	147 (90.18)		
ICU nursing experience (name, %)			*χ* ^2^ = 2.289	0.130
No	18 (11.04)	97 (59.51)		
Yes	21 (53.85)	66 (40.49)		
Job title (name, %)			*χ* ^2^ = 3.327	0.068
Junior	9 (23.08)	63 (38.65)		
Intermediate and above	30 (76.92)	100 (61.35)		
Title (name, %)			*χ* ^2^ = 1.440	0.230
No	37 (94.87)	144 (88.34)		
Yes	2 (5.13)	19 (11.66)		
Length of service (name, %)			*χ* ^2^ = 0.888	0.346
≤ 15 years	25 (64.10)	117 (71.78)		
> 15 years	14 (35.90)	46 (28.22)		
Missed anxiety ($\overset{¯}{X}$ ± *S*, points)	24.44 ± 6.55	21.54 ± 7.16	2.306	0.022^∗^
Emotional exhaustion ($\overset{¯}{X}$ ± *S*, points)	21.67 ± 6.88	16.63 ± 7.04	4.031	< 0.001^∗∗^
Depersonalization ($\overset{¯}{X}$ ± *S*, points)	10.79 ± 4.33	7.14 ± 2.90	6.365	< 0.001^∗∗^
Decreased sense of accomplishment ($\overset{¯}{X}$ ± *S*, points)	21.67 ± 5.12	27.40 ± 5.20	−6.210	< 0.001^∗∗^

^∗^ *p* < 0.05.

^∗∗^ *p* < 0.001.

### 3.2. Scores for Missed Anxiety, Job Burnout, and Job Satisfaction

The missed anxiety score among clinical nurses was 10–42 (22.10 ± 7.12). The total score for job burnout among clinical nurses was 15–77 (51.75 ± 9.58). The mean job satisfaction score was 26–75 (60.28 ± 10.51). Notably, 163 (80.70%) nurses reported moderately high job satisfaction, with a score of 50–75 (64.04 ± 7.72), whereas 39 (19.30%) reported low job satisfaction with a score of 26–49 (44.56 ± 4.08). Table 1 shows the scores of each dimension of the Maslach Burnout Inventory.

### 3.3. Results of Single‐Factor Statistical Analysis of Job Satisfaction

Based on the level of job satisfaction, clinical nurses were categorized into two groups: low‐level and medium‐ to high‐level groups. The results of the single‐factor analysis indicated statistically significant differences in age, missed anxiety, emotional exhaustion, depersonalization, and reduced sense of accomplishment between the two groups (*p* < 0.05). However, no statistically significant differences were observed in terms of sex, marital status, number of children, education level, ICU nursing experience, professional title, position held, or years of service (*p* > 0.05), as presented in Table 1.

### 3.4. Variable Calibration

As the initial sample data did not satisfy fsQCA requirements for Boolean analysis, the condition indices were calibrated using appropriate external standards to convert the original data into values in the [0,1] interval [20]. Continuous fuzzy sets represent the degree to which a condition belongs to a certain set (called membership degree in the QCA method), where 1 represents full membership of the sample and 0 represents no membership. The closer the value is to 1, the higher is the membership degree of the sample and variable. Based on the study by Ketao et al. [7], the intersection of missed anxiety, emotional exhaustion, depersonalization, and reduced sense of accomplishment was at 0.5, with 0.95 defined as full membership and 0.05 as full nonmembership. The calibration information for each condition and the results are listed in Table 2.

**TABLE 2:** Anchor points for variable calibration.

Variable types	Conditional variables	Full membership	Crossing points	Completely unaffiliated
Continuous variables	Missed anxiety	33	22	10
	Emotional exhaustion	30	17.5	6
	Depersonalization	15	6.5	5
	Decreased sense of accomplishment	34	27	17

Dichotomous variables	Age	1	/	0

### 3.5. Individual Condition Necessity Analysis

Before the condition configuration analysis, the “necessity” of each condition was checked one by one. Consistency is an important test standard for the necessary conditions. When the consistency was > 0.9, the condition was considered necessary for the result. Table 3 shows the results of the necessary conditions test for medium to high levels of job satisfaction, analyzed using fsQCA 3.0. Table 3 shows that the consistency level of all the condition variables was < 0.9. Therefore, we observed no necessary condition for a high level of job satisfaction among clinical nurses in this study. Notably, although age showed statistical significance in the univariate analysis (Table 1), its consistency in fsQCA necessity testing was only 0.177, indicating that age alone was insufficient to consistently predict high job satisfaction across all cases. This highlights the advantage of fsQCA in identifying conditional combinations rather than isolated necessary factors.

**TABLE 3:** Analysis of the necessary conditions for a high level of job satisfaction in clinical nurses.

Conditional variables	Consistency	Degree of coverage
Age	0.177	0.707
∼Age	0.823	0.839
Missed anxiety	0.519	0.859
∼Missed anxiety	0.481	0.767
Emotional exhaustion	0.549	0.882
∼Emotional exhaustion	0.451	0.740
Depersonalization	0.633	0.901
∼Depersonalization	0.367	0.694
Decreased sense of accomplishment	0.430	0.712
∼Decreased sense of accomplishment	0.570	0.908

*Note:* ∼ = non.

### 3.6. Configuration Analysis of High Levels of Job Satisfaction

fsQCA3.0 was used to perform the Boolean minimization operation, and the consistency of different variable combinations was analyzed. The consistency threshold was set to 0.75 [21], and complex, intermediate, and simplified solutions were obtained for the medium to high level of job satisfaction of clinical nurses. In general, the intermediate solution performed better than the other two. The core and edge conditions were obtained by comparing intermediate and parsimonious solutions. The agreement between the overall and individual solutions was > 0.80, and the agreement between all solutions met the minimum standard value of 0.75. The consistency and coverage of the overall solutions were 0.900 and 0.750, respectively. The six configurations in Table 4 are sufficient condition combinations for a high level of job satisfaction among clinical nurses. It mainly included (i) Configuration 1: ∼ age ∗ ∼ reduced sense of personal accomplishment, (ii) Configuration 2: missing anxiety ∗ ∼ reduced sense of achievement, (iii) Configuration 3: emotional exhaustion ∗ ∼ reduced sense of accomplishment, (iv) Configuration 4: ∼ age ∗ emotional exhaustion, (v) Configuration 5: depersonalization ∗ ∼ reduced sense of accomplishment, and (vi) Configuration 6: ∼ age ∗ depersonalization. The consistency indices of the solutions for the six configurations were 0.915, 0.903, 0.922, 0.904, 0.918, and 0.908, indicating high consistency. Among them, Configurations 4 and 6 exerted the greatest influence on the medium to high level of job satisfaction. The six configurations jointly explained the main reasons for moderately high levels of job satisfaction among clinical nurses. According to the two‐factor theoretical model framework of job satisfaction of clinical nurses, the six configurations were divided into two types: Configuration 1 was the hygiene‐dominated type, and Configurations 2, 3, 4, 5, and 6 were of the motivation‐health type, as shown in Table 4.

**TABLE 4:** Configuration analysis of medium‐ to high‐level job satisfaction among clinical nurses.

Items	Hygiene‐dominated type	Motivation‐health type				
	Configuration 1	Configuration 2	Configuration 3	Configuration 4	Configuration 5	Configuration 6
Condition variables						
∼Age (moderating variable)	●	—	—	●	—	●
Missed anxiety (H)	—	●	—	—	—	—
Emotional exhaustion (H)	—	—	●	●	—	—
Depersonalization (H)	—	—	—	—	●	●
∼Decreased sense of accomplishment (M)	●	●	●	—	●	—
Pathway characterization	Purely motivator‐dominated type	Hygiene‐motivator hybrid type	Hygiene‐motivator hybrid type	Hygiene‐dominated type	Hygiene‐motivator hybrid type	Hygiene‐dominated type
Theoretical implication	A high sense of achievement (motivator) can independently sustain high job satisfaction	A high sense of achievement (motivator) buffers the negative effects of fear of missing out (FOMO, hygiene factor)	A high sense of achievement (motivator) buffers the negative effects of emotional exhaustion (hygiene factor)	Young nurses exhibit greater resilience to emotional exhaustion (hygiene factor)	A high sense of achievement (motivator) buffers the negative effects of depersonalization (hygiene factor)	Young nurses exhibit greater resilience to depersonalization (hygiene factor)
Consistency	0.915	0.903	0.922	0.904	0.918	0.908
Raw coverage	0.444	0.377	0.408	0.471	0.465	0.526
Unique coverage	0.023	0.008	0.003	0.044	0.014	0.063
Consistency of solutions	0.900					
Coverage of the solutions	0.750					

*Note:* H denotes that the conditional variable is categorized as a hygiene factor in this study, and M denotes the motivator. ● indicates the core condition.

Based on the two‐factor theory framework, six paths were categorized as follows:

Configuration 1 was classified as a “motivator‐dominated path” primarily driven by the positive effect of motivators (high personal accomplishment), with no significant issues related to hygiene.

Configurations 2, 3, and 5 were classified as “hygiene‐motivator hybrid paths,” driven by the co‐occurrence of hygiene factors (e.g., FOMO, emotional exhaustion, and depersonalization) and motivators (high personal accomplishment). These results indicated that high‐level motivators can buffer or offset the negative effects of hygiene factors, thereby sustaining high job satisfaction.

Configurations 4 and 6 were classified as “hygiene‐dominated paths,” driven by a combination of hygiene factors (emotional exhaustion or depersonalization) and youth (age). This suggests that young nurses may maintain a high job satisfaction despite certain work pressures (hygiene‐related issues), potentially because of the career development stage characteristics or stronger psychological resilience.

This classification clearly demonstrates that high job satisfaction among clinical nurses does not depend on a single factor, but rather on complex synergistic or substitutive relationships between motivators and hygiene factors.

## 4. Discussion

### 4.1. Current Level of Job Satisfaction Among Clinical Nurses

In this study, the job satisfaction score of clinical nurses ranged from 26 to 75 (60.28 ± 10.51), indicating a medium to high level of satisfaction. Notably, 163 (80.69%) and 39 (19.31%) nurses were included in the medium to high and low job satisfaction groups, respectively. This result is higher than that reported by Kong [17] on the job satisfaction of clinical nurses in public hospitals in Guangzhou and is consistent with the findings of Deng et al.’s [22] study involving job satisfaction survey of nurses in a Class III Grade A general hospital in Chongqing. The score of burnouts was 15–77 (51.75 ± 9.58), and the score for missed anxiety was 10–42 (22.10 ± 7.12), both of which were at a medium level, consistent with the research results of Kelly and Wang [23, 24]. This may attributed to the fact that clinical nurses included in this study were all from Class III Grade A hospitals, which are more balanced in terms of medical treatment, teaching, and scientific research. In these hospitals, the scale of development and economic benefits are higher than those of other grades of hospitals; therefore, nurses’ welfare is also higher [25]. In addition, these hospitals have more comprehensive human resource management system, providing nurses with greater opportunities to continue education, external training, and career development, enabling them to better realize their personal value, learning, and professional challenges. Therefore, the job satisfaction of these nurses is at a medium to high level.

### 4.2. Configuration Analysis of Factors Influencing Medium to High Level of Job Satisfaction

The fsQCA configuration analysis identified six configurations, belonging to two types. Unlike traditional regression‐based methods, which typically examine the net effects of individual variables in isolation, fsQCA can capture the multifactorial synergy underlying complex organizational phenomena [7, 8]. By adopting a configurational perspective, fsQCA reveals how different conditions combine, rather than compete, to produce an outcome of interest. In this study, the six identified configurations demonstrated that high job satisfaction does not depend on any single factor but rather emerges from specific combinations of motivators and hygiene factors working together. For instance, Configurations 2, 3, and 5 illustrate the buffering synergy between high personal accomplishment (a motivator) and the presence of hygiene‐related challenges (FOMO, emotional exhaustion, and depersonalization).

Notably, our findings provide empirical support for a core tenet of Herzberg’s two‐factor theory: the absence of dissatisfaction does not equate to the presence of satisfaction [9–11]. In this study, the absence of burnout‐related conditions—specifically emotional exhaustion and depersonalization—was neither a necessary condition for high job satisfaction (Table 3) nor a consistent component across all sufficient configurations (Table 4). This aligns with Herzberg’s distinction that hygiene factors primarily serve to prevent dissatisfaction when adequately managed, but their presence does not actively generate satisfaction. Conversely, personal accomplishment—conceptualized as a motivator—appeared as a core or edge condition in all six configurations leading to high job satisfaction. This pattern substantiates Herzberg’s proposition that true job satisfaction results from the presence of intrinsic motivators, not merely the absence of workplace stressors. This distinction exhibits significant practical implications indicating that nursing managers must move beyond simply reducing work stressors (addressing hygiene factors) and proactively cultivate motivators—such as recognition, achievement, and professional growth—to truly elevate job satisfaction.

Among the six configurations, the consistency of the single and overall solutions was higher than the minimum standard of 0.75. The consistency and coverage of the overall solutions were 0.900 and 0.750, respectively, indicating that the configuration conditions in this study were adequate.

#### 4.2.1. Hygiene‐Dominated Path

Hygiene‐dominated type: Configuration 1 (∼age ∗ ∼reduced personal accomplishment): the consistency of job satisfaction among clinical nurses in this configuration was 0.915, with a coverage of 0.444, indicating a relatively high consistency and coverage. A decrease in age and sense of personal accomplishment plays a key role among healthcare clinical nurses, with reduced personal accomplishment representing a healthcare factor. Herzberg’s theory states that when hygiene factors are inadequate, employees may experience unsatisfactory emotions and negative behaviors, such as job burnout and resignation [26]. The Configuration 1 model suggests that nurses would be more satisfied with their work when they have a higher sense of achievement in clinical nursing. Accordingly, nursing managers should provide positive guidance to nurses during their induction training. In daily nursing practice, the work of nurses should be actively encouraged to enhance their sense of achievement, thereby improving job satisfaction and strengthening their efforts to nursing careers. Accordingly, nursing managers and senior hospital leaders should actively listen to the opinions and needs of nursing staff and regularly recognize and reward their achievements to make nurses feel respected and valued. This may enhance their sense of accomplishment and belonging and improve job satisfaction.

The “hygiene‐dominated path” identified in this study is represented by Configuration 1 (∼age ∗ ∼reduced personal accomplishment). Although its conditional combination includes only motivators (high personal accomplishment), personal accomplishment is a core motivator in Herzberg’s two‐factor theory. This path demonstrates that when nurses experience a strong sense of value and recognition of achievement in their work (i.e., motivators are fully satisfied), high job satisfaction can still be attained, even if other hygiene factors (e.g., work pressure and interpersonal relationships) are suboptimal. This aligns with Herzberg’s proposition that motivators exert a direct and positive influence on work‐related attitudes. Therefore, nursing managers should prioritize the establishment of recognition and achievement systems, such as clear performance feedback mechanisms, clinical contribution awards, and professional development pathways, to strengthen this key motivator.

#### 4.2.2. Motivator‐Hygiene Interaction Paths

The “motivator‐hygiene path” identified in this study encompasses five configurations (Configurations 2–6), all characterized by the coexistence and interaction of motivators and hygiene factors within the pathways.

In Configurations 2, 3, and 5, the motivator (high personal accomplishment) emerged as a core condition in combination with distinct hygiene factor issues (FOMO, emotional exhaustion, and depersonalization). This strongly suggests that motivators exert a critical buffering effect; even when nurses experience work environment‐induced stress, anxiety, or alienation (i.e., unmet hygiene factor needs), their overall job satisfaction can still be maintained at a high level if they simultaneously perceive high value and accomplishment in their work. This finding aligns with Herzberg’s original proposition that motivators do not only directly enhance work attitudes; it also extends this by functionally compensating for deficiencies in the hygiene domain, a synergistic dynamic not typically captured in variable‐centered analyses.

In Configurations 4 and 6, youth (aged ≤ 40 years) is combined with hygiene factor issues (emotional exhaustion or depersonalization). This frames youth as a potential protective resource or moderating variable that may render young nurses more resilient to certain occupational stressors. From a developmental perspective, this may reflect differing expectations and tolerance thresholds during the early career stage, where high‐intensity work is often framed as a learning opportunity rather than a chronic stressor [27, 28]. Young nurses may cognitively reappraise emotional exhaustion as an inevitable, even growth‐facilitating challenge, thereby decoupling it from their overall sense of job satisfaction. However, this does not imply that such tolerance is sustainable in the long term; rather, it suggests that the negative effects of hygiene factors may be temporarily buffered by unmeasured motivational factors specific to younger cohorts, such as professional optimism, future‐oriented career expectations, or stronger peer support networks [29].

Beyond the pathways involving younger nurses, Configurations 1, 4, and 6 also feature seniority (age > 40 years, denoted as ∼ age) as a core condition, suggesting that more experienced nurses may achieve high job satisfaction through distinct mechanisms. From a developmental perspective, senior nurses typically occupy a more stabilized career phase than younger nurses, which is characterized by solidified professional identity, clearer role expectations, and accumulated psychological capital, including resilience, self‐efficacy, and adaptive coping strategies developed through years of practice [29]. This accumulated psychological resource enables them to reframe workplace challenges (e.g., emotional exhaustion and depersonalization) as normative occupational experiences rather than threats, thereby decoupling these hygiene factors from overall job satisfaction. Moreover, senior nurses often exhibit greater organizational influence, job autonomy, and access to supportive networks than younger nurses, which can buffer the negative impact of adverse working conditions [27].

However, this apparent resilience should not be misinterpreted as immunity to burnout. Configurations 4 and 6 indicate that even among senior nurses, high job satisfaction can coexist with emotional exhaustion or depersonalization, raising the question of whether such satisfaction reflects genuine well‐being or a form of “adaptive endurance” that masks underlying distress. Longitudinal evidence suggests that prolonged exposure to hygiene‐related stressors, even when buffered by seniority, may eventually erode retention and engagement [23]. Therefore, managerial efforts for senior nurses should focus on strengthening motivators—such as recognition of clinical expertise, opportunities for mentorship roles, and involvement in decision‐making—rather than assuming that satisfaction is inherently stable. By reinforcing intrinsic rewards, managers can help sustain the well‐being of this experienced workforce and prevent latent dissatisfaction from manifesting as turnover.

These findings exhibit actionable implications for nursing management. Optimizing the work environment and reducing workload (addressing hygiene factors) remain essential, while concurrently cultivating motivators. For configurations involving FOMO (Configuration 2), managers should implement information transparency protocols, such as concise digital shift handovers, daily team huddles, or accessible online updates, to reduce professional information anxiety. Additionally, creating structured opportunities for social connection (e.g., peer debriefing sessions and flexible communication channels) can mitigate the social isolation dimension of FOMO.

In contrast, interventions for configurations involving emotional exhaustion (Configurations 3 and 4) and depersonalization (Configurations 5 and 6) should include resilience training programs and peer support groups to buffer the psychological toll of high‐intensity work. Simultaneously, enhancing motivators through autonomy‐in‐practice initiatives (e.g., allowing nurses to lead quality improvement projects or participate in unit‐level decision‐making) may reinforce personal accomplishment.

Notably, for young nurses in hygiene‐dominated paths (Configurations 4 and 6), managers should recognize that while these nurses may currently tolerate certain stressors, they remain at risk for long‐term burnout. Therefore, proactive strategies such as rotation support groups to help them navigate high‐pressure units and psychological capital training (focusing on optimism, self‐efficacy, and hope) can strengthen their coping mechanisms and sustain their engagement over time [30].

In summary, the motivator‐hygiene interaction paths reveal that high job satisfaction is not merely the absence of dissatisfaction, but a dynamic equilibrium where motivators actively buffer hygiene deficiencies. This configurational understanding enables nursing administrators to move beyond generic well‐being programs and design precision interventions targeting the specific combination of factors affecting different nurse subgroups.

#### 4.2.3. Managerial Implications: Tailoring Interventions to Distinct Configurational Pathways

The six configurations identified in this study support a “precision management” approach, suggesting that nursing administrators should move beyond one‐size‐fits‐all well‐being programs and tailor interventions based on the specific pathway a nurse or subgroup predominantly exhibits. Based on the classification in Table 4, we propose three managerial profiles with corresponding intervention strategies: (i) motivator‐dominated path (Configuration 1), (ii) hygiene‐motivator hybrid paths (Configurations 2, 3, and 5), and (iii) hygiene‐dominated paths with youth (Configurations 4 and 6).

Nurses within Configuration 1 sustain high job satisfaction primarily by a strong sense of personal accomplishment, a core motivator, without significant hygiene‐related issues. Therefore, managers should focus on sustaining and amplifying intrinsic motivators. Recommended interventions include (i) implementing structured recognition programs (e.g., “clinical excellence awards” and “nurse of the month”) to publicly acknowledge achievements; (ii) providing regular, constructive feedback that reinforces competence and contribution; and (iii) offering opportunities for professional development (e.g., specialty certifications, leadership training, and involvement in quality improvement projects) to maintain a sense of growth and challenge. Notably, interventions focusing primarily on reducing hygiene factors (e.g., workload reduction) may yield diminishing returns for this group. Consequently, managerial resources should be directed toward enriching the work itself.

High job satisfaction among nurses within Configurations 2, 3, and 5 coexists with the presence of specific hygiene factor challenges (FOMO, emotional exhaustion, or depersonalization), buffered by high personal accomplishment. Therefore, a dual‐focused strategy is required: concurrently enhancing motivators while addressing the specific hygiene factor at play. For Configuration 2 (FOMO), managers should improve information transparency and social connection. Specific interventions include implementing concise digital shift handovers to ensure critical updates are accessible to all; conducting daily team huddles to disseminate key information; creating structured opportunities for peer connection (e.g., debriefing sessions and informal check‐ins) to mitigate social isolation; and ensuring equitable access to training and promotion information. For Configuration 3 (emotional exhaustion), managers should implement workload management and resilience‐building strategies. Recommended interventions include reviewing shift patterns to prevent excessive consecutive shifts; providing access to employee assistance programs; offering resilience training workshops focused on stress management and self‐care; and fostering a supportive unit culture where nurses feel comfortable discussing workload concerns. For Configuration 5 (depersonalization), managers should focus on rebuilding relational connections and restoring meaning in work. Recommended interventions include facilitating peer support groups or mentoring programs; organizing structured debriefing sessions after challenging clinical events; recognizing compassionate care as a valued competency; and involving nurses in patient‐centered initiatives that reconnect them with the purpose of their work.

Young nurses (aged ≤ 40 years) within Configurations 4 and 6 achieve high job satisfaction despite the presence of emotional exhaustion or depersonalization. While these nurses may currently demonstrate resilience, this pattern also signals potential vulnerability. Without sustained motivators, such tolerance may erode over time, increasing the risk of eventual burnout. Therefore, managers should adopt a proactive, developmental approach rather than assuming these nurses are “fine” based on their current satisfaction levels. Recommended interventions include (i) mentorship programs pairing young nurses with experienced mentors to provide guidance, emotional support, and career navigation; (ii) psychological capital training focusing on self‐efficacy, optimism, hope, and resilience to strengthen coping resources; (iii) career counseling to help young nurses articulate long‐term professional goals, transforming temporary workplace stress into perceived career investment; and (iv) regular check‐ins with supervisors to monitor well‐being and identify early signs of burnout before they become entrenched. Notably, managers should ensure that these young nurses also have access to motivators (e.g., recognition and growth opportunities) to prevent the current hygiene‐dominated pattern from becoming a long‐term vulnerability.

Hence, by moving beyond one‐size‐fits‐all well‐being programs and implementing pathway‐specific interventions, nursing managers can more effectively allocate resources, address root causes, and foster a more resilient and satisfied nursing workforce. This configurational approach enables managers to distinguish between nurses who require hygiene‐focused support, those who need motivator enrichment, and those who need both—ultimately enhancing both individual well‐being and workforce stability.

### 4.3. Limitations and Future Research

Despite the positive outcomes, this study has some limitations. First, the cross‐sectional design precludes causal inferences, and the configurational pathways identified represent associations rather than deterministic relationships. Second, the single‐center sample from a tertiary hospital in Beijing limits the generalizability of the findings to broader nursing populations. Notably, the participants were recruited from a blood purification center—a specialized unit characterized by high patient acuity, intensive technical demands, shift‐based schedules, and high workloads. While this specific context may enhance the internal validity of the findings for similar high‐acuity units (e.g., ICUs, emergency departments, and dialysis centers), it may also restrict generalizability to nurses in general wards, community health centers, or institutions in different cultural and socioeconomic contexts, where workload patterns, professional resources, nurse demographics, and stress profiles may differ significantly. For instance, nurses in general wards may experience different types of stressors (e.g., patient turnover and family communication) compared to the technical and life‐support demands common in blood purification settings. Third, although the FOMO scale demonstrated good psychometric properties, it was originally designed for social media contexts. Therefore, future research should consider developing or adapting instruments that more precisely capture profession‐specific information anxiety and social isolation in clinical nursing environments. Finally, although our model achieved high coverage, other unmeasured variables, such as organizational culture, leadership style, psychological capital (e.g., resilience and optimism), and family support, may also influence these configurational pathways and should be explored in future studies. Consequently, multicenter, cross‐regional, and longitudinal studies are required to validate and extend our findings.

## 5. Conclusion

This study advances nursing management theory by applying fsQCA method to uncover the configurational pathways through which Herzberg’s two‐factor theory operates in clinical nursing. The findings demonstrate that high job satisfaction results from specific combinations of motivators (e.g., personal accomplishment) and hygiene factors (e.g., emotional exhaustion, depersonalization, and FOMO), rather than from any single determinant. In this study, we identified six sufficient configurations, which were categorized into motivator‐dominated, hygiene‐motivator hybrid, and hygiene‐dominated types, illustrating that synergistic interactions, rather than isolated factors, shape outcomes.

The key theoretical contributions of this study are twofold. First, the study reveals the buffering role of personal accomplishment, a core motivator, which can offset the negative effects of unmet hygiene needs. Second, it identifies age as a contextual moderator, with younger nurses exhibiting distinct pathways where hygiene factors coexist with high satisfaction, suggesting developmental differences in stress appraisal and coping resources. These findings extend the two‐factor theory by specifying the configurational logics through which its components combine in real‐world settings, moving beyond variable‐centered approaches to capture multifactor synergy.

Based on these configurational findings, the study provides an evidence‐based framework for designing targeted, pathway‐specific interventions, such as achievement recognition programs for motivator‐dominated paths, dual‐focused strategies for hybrid paths, and resilience support for young nurses in hygiene‐dominated paths, enabling hospital administrators to move beyond one‐size‐fits‐all approaches. Ultimately, this configurational perspective offers a holistic understanding of nurse job satisfaction and practical pathways for enhancing workforce stability and well‐being.

## Funding

This research did not receive any specific grant from funding agencies in the public, commercial, or not‐for‐profit sectors.

## Conflicts of Interest

The authors declare no conflicts of interest.

## Data Availability

The datasets generated and analyzed during the current study are not publicly available due to privacy and confidentiality agreements with participants but are available from the corresponding author on reasonable request and with appropriate ethical approvals.
